# Melatonin and Depression: A Translational Perspective From Animal Models to Clinical Studies

**DOI:** 10.3389/fpsyt.2021.638981

**Published:** 2021-04-08

**Authors:** André C. Tonon, Luísa K. Pilz, Regina P. Markus, Maria Paz Hidalgo, Elaine Elisabetsky

**Affiliations:** ^1^Laboratório de Cronobiologia e Sono, Hospital de Clínicas de Porto Alegre, Porto Alegre, Brazil; ^2^Graduate Program in Psychiatry and Behavioral Sciences, Federal University of Rio Grande do Sul, Porto Alegre, Brazil; ^3^Laboratório de Cronofarmacologia, Departamento de Fisiologia, Instituto de Biociência, Universidade de São Paulo, São Paulo, Brazil; ^4^Programa de Pós-Graduação em Ciências Biológicas-Bioquímica, Departamento de Bioquímica, Universidade Federal do Rio Grande do Sul, Porto Alegre, Brazil

**Keywords:** psychiatry, mood disorder, neuropsychaitric disorders, behavior, biological rhythms, chronobiology, 6-sulfatoxymelatonin (aMT6s), biomarker

## Abstract

Daily rhythm of melatonin synchronizes the body to the light/dark environmental cycle. Several hypotheses have been raised to understand the intersections between melatonin and depression, in which changes in rest-activity and sleep patterns are prominent. This review describes key experimental and clinical evidence that link melatonin with the etiopathology and symptomatology of depressive states, its role in the follow up of therapeutic response to antidepressants, as well as the clinical evidence of melatonin as MDD treatment. Melatonin, as an internal temporal cue contributing to circadian organization and best studied in the context of circadian misalignment, is also implicated in neuroplasticity. The monoaminergic systems that underly MDD and melatonin production overlap. In addition, the urinary metabolite 6-sulfatoxymelatonin (aMT6) has been proposed as biomarker for antidepressant responders, by revealing whether the blockage of noradrenaline uptake has taken place within 24 h from the first antidepressant dose. Even though animal models show benefits from melatonin supplementation on depressive-like behavior, clinical evidence is inconsistent vis-à-vis prophylactic or therapeutic benefits of melatonin or melatonin agonists in depression. We argue that the study of melatonin in MDD or other psychiatric disorders must take into account the specificities of melatonin as an integrating molecule, inextricably linked to entrainment, metabolism, immunity, neurotransmission, and cell homeostasis.

## Introduction

Melatonin (N-acetyl-5-methoxytryptamine) is an amphiphilic indoleamine conserved from unicellular organisms to plants and animals. In mammals, pineal and extra-pineal synthesis were described (1). Darkness at night triggers a polysynaptic pathway that begins with the retino-hypothalamic tract projecting to the suprachiasmatic nuclei (SCN) of the hypothalamus and ends as a sympathetic input to the pineal gland. Melatonin synthesis is prompted by norepinephrine and requires serotonin as a precursor (2). Pineal melatonin, a primary output of the circadian pacemaker (SCN), transduces light-dark information to the whole body, coordinating daily physiological functions and behaviors.

**Figure 1:**
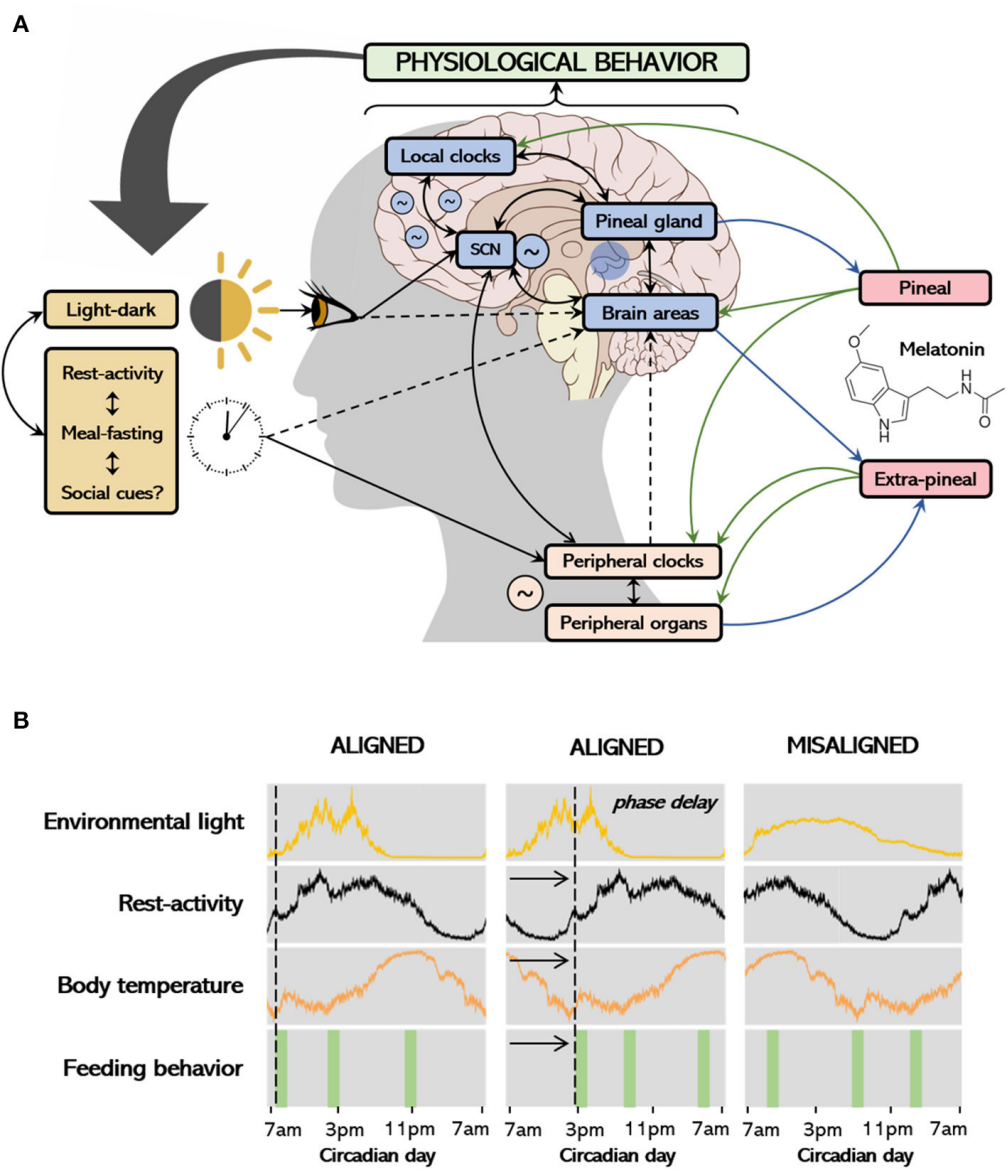
Environmental and internal regulation of biological rhythms, melatonin production and associations with behavior **(A)**; the process of entrainment and alignment **(B)**. The main environmental cue that synchronizes the circadian system is the light-dark cycle. The suprachiasmatic nucleus (SCN) receives photic information and projects to several structures in the body, coordinating peripheral tissues by modulating other oscillators according to light-dark transitions. Other stimuli of importance include feeding schedules, rest-activity rhythm and social cues. Peripheral tissues respond to timing neural and endocrine signals modulated by the SCN, including the release of melatonin to the bloodstream, as well as from feeding. On the other hand, endogenous circadian rhythms also influence daily patterns in behavior/exposure to environmental factors and might also influence melatonin production (see details in text). In **(A)**, black arrows connect the environmental stimuli to the central and peripheral clocks and tissues and show the interconnection among systems. Continuous arrows represent higher levels of evidence and dashed arrows represent lower levels of evidence. Melatonin is produced in the pineal gland, as a result of darkness or on-demand via other pathways (see details in text), acting locally or transducing the dark signal to peripheral tissues (green arrows). Extra-pineal sources of melatonin include the skin, guts, and lungs, which only act locally with no known chronobiotic effect. While the physiological actions of melatonin potentially impact behavior, individual behavior might also determine the timing of light exposure, rest-activity rhythms and feeding behavior, thus regulating melatonin secretion. The first column at **(B)** shows rhythms of activity, temperature, and feeding behavior aligned with the light signal; the second depicts rhythms which are phase delayed. The rhythms at the third column are no longer entrained to the light signal and are not synchronous within themselves, determining a misalignment.

Biological rhythms and melatonin represent recent frameworks in the investigation of the etiology, prognostic improvement, and treatment approach in the clinical course of depression. The somatic, cognitive, and affective symptoms of depressive states that occur in association with neurochemical/hormonal imbalance, may result, among other factors, from the misalignment of biological rhythms (3–5). A summary of the physiological regulation and organization of biological rhythms is shown in Figure 1.

Considering the cyclic nature of mood disorders, the shared monoaminergic involvement, the data from seasonal depression, and the marked sleep and circadian changes in depressive disorders, several hypotheses have been raised to understand the intersections between melatonin with depression. Experimental and clinical studies were designed to understand possible pathophysiological connections and devise therapeutic strategies to optimize the pharmacological management of depressive patients. This review aims to discuss the interconnections of melatonin production pathways and its physiological actions with the etiopathology and clinical approaches to depression. We will initially describe the framework of depression relevant for the understanding its association with biological rhythms and melatonin. We proceed by discussing the role of melatonin as an internal temporal cue, its production and physiological actions, and its role as a neurohormone sharing common neuro-endocrine inputs with the pathways associated with depression. Finally, we describe the current knowledge from clinical research in the field, including the use of exogenous melatonin and urinary 6-sulfatoxymelatonin as treatment and response biomarker, respectively.

## Depression and Biological Rhythms

### What Do we Mean by Depression?

Depression is a term with multiple meanings. As a state, a depressive mood may be an adaptive stress response (6). Depressed mood states manifest as typical behavioral phenomena (e.g., anhedonia, psychomotor disturbance) and dysregulation in neurovegetative functions (e.g., altered appetite, disturbed sleep), which may be a result of a great variety of physiological events. As part of a mental disturbance, depressive symptoms can manifest in several conditions such as unipolar or bipolar mood disorder, substance abuse, and general medical disease. On the other hand, Major Depressive Disorder (MDD), as defined by international guidelines such as the Diagnostic and Statistical Manual of Mental Disorders (DSM) and the International Classification of Diseases ICD, is a syndromic construct assembled as a distinct clinical condition (7, 8). According to the fifth edition of the DSM, at least one of the two cardinal symptoms must be manifested to reach a positive diagnosis: depressed mood and/or loss of interest or pleasure in daily activities. Of the nine cognitive (e.g., impaired concentration), emotional (e.g., feelings of worthlessness) and somatic symptoms (e.g., psychomotor agitation or retardation, changes in appetite, sleep disturbances) listed as diagnostic criteria, at least five must be present during the same 2-week period, with the symptoms representing a change from previous baseline. Of note, other medical conditions that can cause the same symptoms must be excluded. MDD is one of the most burdensome disorders and the leading cause of disability worldwide (9, 10). Therefore, the assessment of the severity of the depressive syndrome (as a continuous feature) in clinical and research settings is fundamental, which typically implies the use of validated self-reported instruments and structured interviews of cognitive, physical, and psychomotor changes. Though sadness and anhedonia are the cardinal symptoms of MDD, the multivariate features of depression, together with its multifactorial etiology and the lack of a reliable biomarker, can explain the individual differences in pharmacological response.

Clinical response is usually noticeable after 2–5 weeks (11–13). The poor or absent response to antidepressants that is associated with the morbidity and mortality of MDD justifies the search for improvement in diagnosis, while searching for subtypes of depression that show treatment idiosyncrasies. In addition, preventing the relapse and recurrence of clinically significant symptoms is essential for new treatment approaches and/or the identification of markers of response to established treatments. In fact, several studies document a vast heterogeneity of clinical features of depressive disorders, indicating that specific symptoms represent different physiological phenomena, boosting the search for specific diagnostic and treatment biomarkers and unraveling new therapeutic strategies (14). In this context, despite inherent limitations, animal models have been instrumental in the investigation of subjacent neuronal mechanisms associated with the genesis, trigger factors, and maintenance of depressive states, as well as potential treatments.

The difficulties in modeling depression and neuropsychiatric disorders in experimental non-human species may, in part, be attributed to the variability of depressive symptoms (across and within individuals), and the sheer impossibility to reproduce in non-human animals some important features, such as guilt and suicidality. This somehow mirrors the challenges of Psychiatry as a field in categorizing debilitating disorders (with overlapping symptoms and varied underlying mechanisms) as opposed to normal behavior. Chronic stress, maternal deprivation, and olfactory bulbectomy are among the models that present higher degrees of face validity (i.e., animal behavior correlates with MDD symptomatology), predictive validity (i.e., good chances of replicating the positive or negative results in human patients), and construct validity (i.e., consistent underlying pathology biomarkers, triggering factors, time course, and response to antidepressants). The majority of animal models only replicate anhedonia (with varying degrees of face value), which is certainly one of the core symptoms of depression. The widely used tail suspension and forced swimming tests have no pathophysiological or undisputed behavioral manifestations equivalent to those seen in depression but possess some degree of translation predictability. It is of utmost importance to select the model appropriate for a given question, and interpret results considering specific limitations.

### Are Biological Rhythms Related to Depression?

Although the challenges of assessing behavior pose inherent limitations to both experimental and clinical studies, the relationships between depressive-like behavior and biological rhythms have been abundantly documented. Nile grass rats (*Arvicanthis niloticus*), a diurnal rodent, exposed to short photoperiod (5 h light/19 h dark) for 6 weeks presented reduced saccharin preference, higher immobility time in the forced swim test, and no changes on time spent in the light side of the dark/light box in comparison to controls (15). The same species submitted to low light intensity during the daytime (cycles of dim light of 50 lux during 12 h/dark during 12 h) for 4 weeks showed increased immobility and decreased climbing in the forced swim test; again, no effects were observed in the light/dark test or locomotion in the open field compared to bright light controls. Nile grass rats also show lower dopaminergic and somatostatin neurons in the hypothalamus under both short photoperiods and dim light/dark cycle (16). In nocturnal mice, dim light at night [16 h of light (~150 lux) and 8 h of darkness (5 lux)] for 4 weeks lead to depression-like behaviors such as increased immobility in the forced swim test and reduced sucrose preference (17). In addition, nocturnal rodents under constant light for 3–4 weeks, a light regimen known to render rats behaviorally arrhythmic (18), exhibited significant higher number of grooming events during the open field test, and diminished sucrose preference throughout the study (19). Under forced desynchrony (light/dark cycles of 22 h), a protocol known to uncouple neuronal oscillators in the suprachiasmatic nucleus (SCN; see Figure 1), rats develop into depressive-like phenotypes (i.e., anhedonia, sexual dysfunction, increased immobility in the forced swim test) (3).

Landgraf et al. (4) showed that knocking down *Bmal1* (one of the essential clock genes that participate in the maintenance of the circadian periodicity at the cellular level) in mice suprachiasmatic nucleus (SCN) led to increased escape latencies and number of escape failures in the learned helplessness paradigm, induced higher immobility time in the tail suspension test, and decreased the time spent in the light compartment of the light/dark test. These results indicate that disrupting central SCN rhythms causes helplessness, behavioral despair, and anxiety-like behavior. The experiment supports a causal relationship because environmental light-dark cycles and light input pathways were unchanged. Therefore, results cannot be explained by light affecting depressive-like behavior through other pathways [i.e., without disturbing sleep or circadian rhythmicity, or through pathways unrelated to the SCN (20)]. Unlike in global knockouts in which mice may also present altered behavior related to pleiotropic functions of the clock genes, the model has the advantage of selectively inducing disturbed SCN rhythms while avoiding other neural damages caused by SCN lesions. Moreover, hedonic behavior measured by the sucrose preference test, spatial preference in the open field test, and aversion to eating in a novel environment were unchanged, suggesting that only specific aspects of depressive-like behavior are influenced by the disruption of the central clock (4). Of note, neurotransmitter systems altered in depression show blood levels circadian oscillations (21, 22), though it is unclear whether or how mood states could affect this circadian pattern.

Clinical evidence supports that dysfunctions of the circadian timekeeping system are present in a variety of morbid clinical conditions, such as obesity, diabetes, hypercholesterolemia, cardiovascular diseases, and cancer (23, 24). Mood alterations have also been extensively studied in models of circadian disruption or misalignment (see Figure 1). Of relevance to this discussion, depressive symptomatology can assume seasonal variation (i.e., seasonal affective disorder), and is associated with non-respiratory sleep disturbances and distinguishable daily motor activity patterns (i.e., comparing depressed individuals with non-depressed individuals and among depressive subtypes). Diurnal variations in mood have been seen in naturalistic conditions (25) and experimental results suggested mood to be affected by an interaction between circadian phase and duration of prior wakefulness (26). A few initiatives arouse in the past decade aiming to elucidated whether altered patterns of diurnal variations in mood are associated with depressive disorders. These reports show that individuals with depression are more likely to report diurnal variations in mood (27, 28).

Acute or chronic dysfunctions of the circadian timekeeping system (e.g., Eastbound travels across time-zones and rotating night shift workers, respectively) impact the organism with consequences at different biological scales, i.e., cellular, tissue, and systemic level. In the light of the above, circadian misalignment can result from: (1) damage to cerebral structures like the retina, the retino-hypothalamic tract or the SCN; (2) genetic manipulation or variations in *clock* and *clock-controlled* genes; (3) a response to external factors (*zeitgebers*) in situations of rotating night shift work, travel across time zones, lack of light exposure during daytime or the excessive exposure during the night. All these potentially impact the production of pineal melatonin, an important temporal cue of biological rhythms.

## Melatonin: The Neuroendocrine Timing Signal

The main environmental cue that synchronizes biological rhythms with the environment is the light-dark cycle. The SCN, a small collection of hypothalamic cells just above the optic chiasm, receives environmental photic information collected by intrinsically photosensitive ganglion cells (ipRGC) in the retina. The ipRGC expresses the photopigment melanopsin, which transduces light wavelengths into neural input through the retino-hypothalamic tract to the SCN. The SCN projects to several structures, including the paraventricular hypothalamic nucleus that communicate with the intermediolateral spinal column (29). The light/dark information reaches the pineal gland through sympathetic postsynaptic fibers of the superior cervical ganglion (30), and melatonin is simultaneously released to the peripheral circulation and to the cerebrospinal fluid (CSF) (31). It is the daily pattern of melatonin secretion that carries information for circadian and seasonal temporal organization (32). Melatonin is also synthesized by the skin, guts, and lungs in a constitutive manner, and on-demand by activated immune-competent cells, such as monocyte-derived and resident macrophages, microglia and lymphocytes (1, 33, 34).

It is currently impossible to monitor the central clock (SCN activity) directly in humans. Since the timing of melatonin secretion is strongly regulated by the SCN, the onset of melatonin secretion under dim light (dim light melatonin onset; DLMO) has been considered a gold standard to assess the central clock's phase and whether it is misaligned in relation to other internal rhythms or environmental cycles. A fair number of studies have investigated the relationship between depressive symptoms and the phase angle difference between DLMO and the timing of other rhythmic functions, including sleep (35, 36). Results are heterogeneous, which may reflect the multifactorial nature of depression and the multiple actions and regulatory systems related to melatonin. Aside from phase shifts, lower nocturnal melatonin levels are often, though not always, reported in depressed individuals (37–39).

Even though the molecular and mechanistic underpinnings are yet to be unveiled, the way the clock, sleep and behavior co-exist and co-affect each other seems crucial to balance health and disease [see (40) for discussion]. In the context of mood disorders, we still need to identify sub-optimal phase relationships and other altered circadian states that may affect the susceptibility to develop specific depressive-like behaviors. Melatonin—as the important phase marker and *eigen-zeitgeber* it is—is likely to remain a central player in this framework.

## Melatonin Production and Physiological Actions

### What Is the Pathway of Melatonin Production?

**Figure 2:**
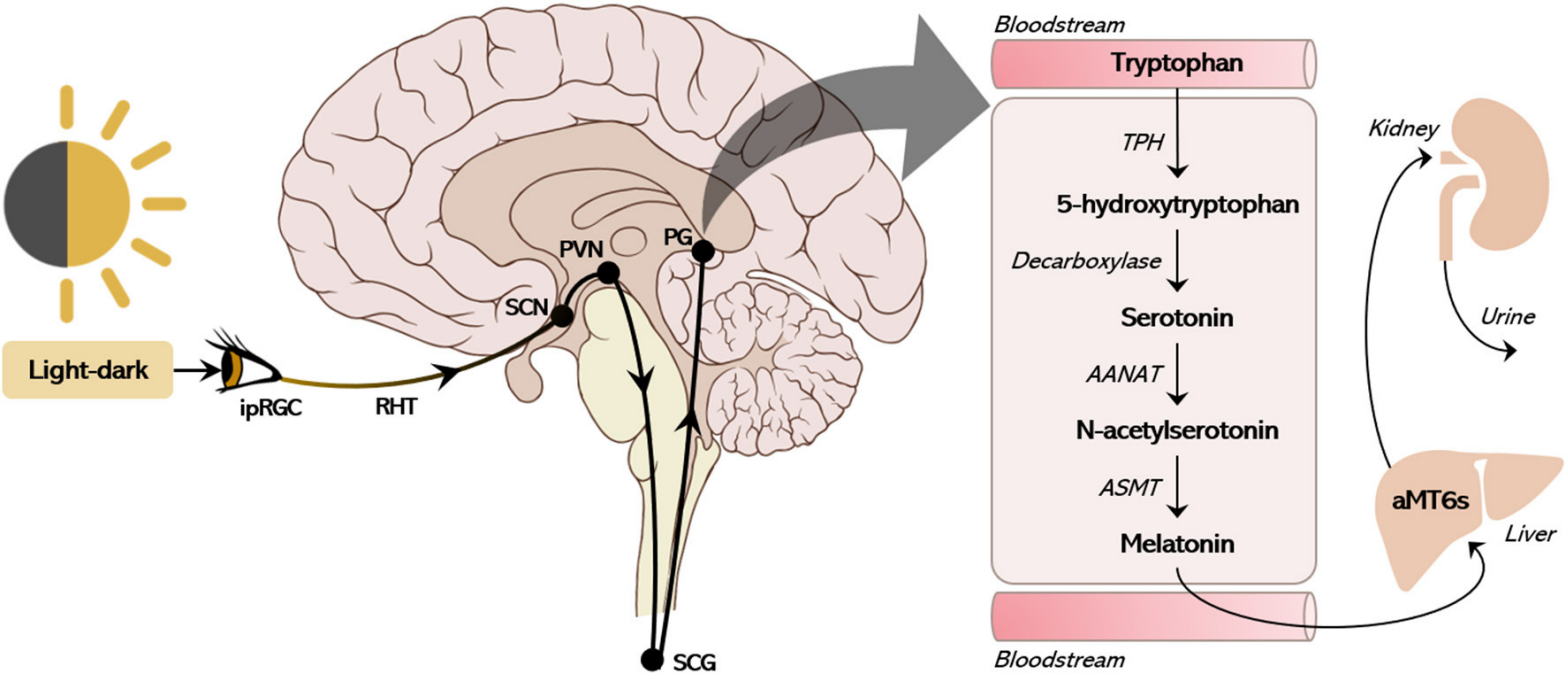
The canonical pathway of pineal melatonin production. The suprachiasmatic nucleus (SCN) receives environmental photic information collected by intrinsically photosensitive ganglion cells (ipRGC) in the retina. The ipRGCs express the photopigment melanopsin, which transduces light wavelengths into neural input through the retinohypothalamic tract (RHT) to the SCN. The SCN constitutively inhibits the hypothalamic paraventricular nucleus (PVN) via GABAergic projection. In the absence of light, the PVN activates the ganglion cervical nuclei (SCG, via the intermediolateral column of the medulla) triggering noradrenergic fibers that innervate the pineal gland (PG), ultimately releasing the co-transmitters noradrenaline and ATP. This sympathetic stimulus triggers the action of arylalkylamine N-acetyltransferase (AANAT) converting serotonin (5TH) into N-acetylserotonin (NAS) within the pinealocyte. With the constitutive action of N-acetylserotonin O-methyltransferase (ASMT), NAS is then converted to melatonin and immediately released into the cerebrospinal fluid and bloodstream. Through first-pass metabolism in the liver, melatonin is converted to 6-sulfatoxymelatonin (aMT6s), which is then excreted in the urine.

In mammals, the biosynthesis of melatonin starts with the conversion of tryptophan to l-5-hydroxytryptophan, which is converted to serotonin [5-hydroxytryptamine(5-HT)] by the aromatic l-amino acid decarboxylase (AADC). Serotonin is acetylated by the phosphorylated arylalkylamine N-acetyltransferase (P-AANAT), forming N-acetylserotonin (NAS), which is converted to melatonin by N-acetylserotonin O-methyltransferase (ASMT). The melatonin pineal daily rhythm is determined by the conversion of 5HT to NAS under sympathetic control (Figure 2). Environmental light modifies the structure of melanopsin in the retinal ganglion cells triggering glutamate excitation at the retino-hypothalamic tract that projects to the SCN. The SCN inhibits the hypothalamic paraventricular nucleus (PVN) via GABAergic projection. In the absence of light, the PVN activates the ganglion cervical nuclei (via the intermediolateral column of the medulla) activating noradrenergic fibers that innervate the pineal gland, ultimately releasing the co-transmitters noradrenaline and ATP (41). Consequently, the production of melatonin depends on the integrity of the brain monoaminergic system (2). Beta-1 adrenergic receptor activation leads to increase in cAMP and activation of protein kinase A, which promotes the phosphorylation of the cyclic AMP regulating element (CREB) and the phosphorylation of AANAT (P-AANAT). Phospho-CREB induces the transcription of the gene that codifies AANAT (the native form of the enzyme), which is immediately degraded by the proteasome. In nocturnal animals both the control of transcription and activation of AANAT plays an important role in melatonin synthesis, while in diurnal animals the transcription of the gene is constitutive (1); thus, nocturnal melatonin surge is delayed in nocturnal rodents when compared to humans. Circulating melatonin levels in the bloodstream accurately reflects pineal synthesis (2). At dark-night the pineal melatonin plasma levels show a 10–20-fold increase in normally entrained individuals. When light is turned on in the middle of the dark night, the pineal melatonin synthesis stops, and melatonin concentration in the blood is abruptly reduced due to liver metabolization (first-pass effect). In the liver, melatonin is metabolized to 6-sulfatoxymelatonin (aMT6s), excreted in the urine, which is well-correlated with plasma melatonin (Figure 2) (42).

### How Is Melatonin Production Regulated?

The pineal gland is a circumventricular organ that monitors the level of pathogen-associated molecular patterns (PAMPs) and damage-associated molecular patterns (DAMPs) in the cerebrospinal fluid (CSF) and peripheral circulation. In pinealocytes, the activation of the NFκB pathway by toll-like receptors and TNFRs inhibits AANAT-gene transcription both in diurnal and nocturnal animals. Cortisol or corticosterone at concentrations compatible with arousal (but not the ones found in models of chronic unpredictable mild stress) potentiate melatonin synthesis, while concentrations compatible with immune suppression blocks it. In immune-competent cells PAMPs and DAMPs promote the synthesis of melatonin by activating the transcription of the gene that codifies AANAT. This process is also dependent on the binding of NFkB dimers to the gene promoter, but it is not an immediate effect. Activation of PAMPs or DAMPs receptors promote the nuclear translocation of the NFkB dimer (p50/p65) that leads to the synthesis of the subunit (cRel) necessary for activating AANAT transcription. The induction of melatonin synthesis in rodents and human macrophages by the transcription factor is a hallmark in inflammatory responses and inflammation (43–46). The fact that stressful conditions increase extra-pineal melatonin synthesis independently of environmental light strongly suggests that the amplitude of daily melatonin rhythm, classically attributed to reduction of nocturnal melatonin synthesis, may also result from an increase of the output from extra-pineal sources (1).

During the mounting of an innate immune response melatonin plays various roles: the reduction of nocturnal melatonin leads to the mobilization of leukocytes from the bone marrow to the blood, and from the blood to the site of lesion; the synthesis of melatonin by macrophages/microglia increases its phagocytic ability and reduces the oxidative stress participating in the recovery phase of the inflammatory response (1). The return of macrophages/microglia/phagocytes counts to baseline requires the return of mediators' levels active at the recovery phase, including melatonin, to basal values. When the recovery from the acute inflammatory response is mediated only by pineal activity return to baseline, without restoration of macrophage melatonin synthesis to baseline, the result is a resilient state (low-inflammatory grade).

Over the past decades, studies reported neuroinflammatory responses in a series of neurodegenerative and psychiatric disorders. Intense research has been undertaken to determine the critical elements of such responses and putative therapies for their suppression. Some reports associate MDD to increased levels of cytokines including TNFα, IL-6, and IL-1β, as well as a reduction in complement C3 (47–49). Increased immune-inflammation, with high oxidative and nitrosative stress leading to changes in neuronal regulating tryptophan catabolites (TRYCATs) and mitochondrial dysfunction, have been documented in the progressive course of MDD (50, 51). In animal models, melatonin treatment significantly abolished the effects of LPS and reduced NF-κB in the cortex and the hippocampus, both effects resulting in an improvement of depressive-like behaviors (52). These results point to the possibility of an “antidepressant effect” of melatonin via the interplay with the immune system. Furthermore, as described above, cortisol functioning and its circadian fluctuation is essential for the adequate melatonin surge. Blunted cortisol rhythms (i.e., lower morning cortisol peak and higher daytime values) have been demonstrated in association with depressive symptoms in humans (49, 53, 54).

### How Does Melatonin Exert Its Physiological Actions?

Melatonin effects triggered by a large range of concentrations (pM to mM) are mediated by mechanisms of action with different grades of sensitivity. The effects of exogenous vs. endogenous melatonin, as well as the concentration reached by intracrine/autocrine, paracrine and hormonal sources, depend on the melatonin bioavailability and the mechanisms that mediate each production source. In a concentration range compatible with the nocturnal surge (pM to nM), melatonin orchestrates a plethora of physiological functions by activating GPCRs receptors (MT1 and MT2) localized in plasmatic, nuclei and mitochondrial membranes. At much higher concentrations, melatonin may act as an electron donor, promoting a receptor free non-specific antioxidant response (55). Other non-receptor mediated actions include the regulation of clock genes expression (by directly inhibiting the proteasome), and inhibition of the ubiquitin–proteasome system that ultimately controls protein degradation.

Melatonin receptors MT1 and MT2 couple to Gi/o and beta-arrestins and depending on the context also couple to Gq (55). The second messenger immediate responses result in an increase/decrease of cAMP or increase in intracellular or intramitochondrial free calcium (56). The internalization of the receptors upon stimulation leads to the activation of ERK pathways, which modulate intranuclear responses. MT1/MT2 receptors form heterodimers with pharmacological properties different from MT2 homodimers. The heterodimerization of the MT1 receptor with the orphan receptor GPR50 impairs its interaction with melatonin (57). Thus, another source of variability of melatonin response are the changes in the expression and the dimerization of melatonin receptors induced by pathophysiological and pathological states.

Pineal melatonin exerts a wide range of physiological actions due to its release into the cerebrospinal fluid (CSF) and bloodstream. As a hormone that gains the bloodstream at night in the dark, melatonin is an internal temporal cue that has immediate interactions with its molecular effectors as well as prospective effects, since it primes physiological responses that will take place hours after its peak. The duration, timing, and cyclic-nature of pineal melatonin production, as well as its seasonal variation according to photoperiod changes, contribute to the temporal organization of physiological phenomena into circadian and circannual timescales (58). Finally, transgenerational effects have been described, as maternal melatonin reaches the fetus via placenta and constitutes the only fetal source of intrauterine melatonin. As previously stated, extra-pineal melatonin shows immediate effects in the cells and tissues where it is produced, and the current knowledge does not support any *eigen-zeitgeber* action (i.e., being an internal temporal cue) or seasonal effect resulting from these sources. A detailed review on the physiological actions of melatonin was composed by Cipolla-Neto and Amaral (59).

## Melatonin and Depression: Is There a Physiological Overlap?

### How Does Melatonin Relate to the Monoamine and Neurotrophic Hypotheses of Depression?

Since the report of two pharmacological agents in the late 1950s, the pathophysiology of MDD has been linked to a depletion in monoaminergic neurotransmitters, such as noradrenaline, serotonin and dopamine (5, 60). Initial studies showed that severe depressive episodes could be effectively treated with monoamine oxidase inhibitors (MAOi) and tricyclic antidepressants (TCA), which share increased availability of brain catecholamines as a common pathway. The involvement of other monoamines has been studied, with growing evidence to the role of serotonin with the introduction of Selective Serotonin Reuptake Inhibitors (SSRIs) in the 1960s.

Since melatonin production occurs from serotonin after stimulation of adrenoreceptors (see section What Is the Pathway of Melatonin Production? above), it has been hypothesized that a disturbance in melatonin secretion could be present in the acute phase of depressive illness, and possibly be related to its pathophysiology. Moreover, melatonin controls dopamine signaling in the forebrain, hypothalamic and hippocampal areas (61–63). Of note, dopamine is not only the precursor of noradrenaline, but also has been implicated in the circadian regulation of melatonin production (64), via activation of dopamine D4 receptors by noradrenergic signals (65).

Several clinical studies tried to identify alterations in absolute levels of melatonin as a possible maker of depressive states; considering the above detailed physiological regulation of melatonin, this was a misleading proposition, and the evidence is at best controversial. While some earlier studies reported lower nocturnal serum or saliva levels of melatonin in depressed individuals, others reported no differences, or even higher levels compared to controls (39). One study (38) reported that individuals with levels of melatonin in the two lowest quartiles had higher risk of severe symptomatology; nevertheless, the authors described similar levels of nocturnal melatonin in depressed patients and controls. Studies that investigated the nocturnal production of 6-sulfatoxymelatonin (aMT6s) found no difference between depressed individuals and controls before treatment (66, 67), suggesting the absence of a linear relationship between depressive behavior and melatonin levels. One study (37) found lower bedtime levels of plasma melatonin in depressed individuals with melancholic features; others (68, 69) indicated that subgroups of depressed individuals who do not suppress morning cortisol after dexamethasone stimulation test are more likely to present lower nocturnal melatonin. These data indicate that the lack of agreement in findings on melatonin levels in depressed individuals may be a result of (1) the diversity of behavioral and etiological phenomena in depressive states [as previously commented (14)]; (2) methodological heterogeneity, like inconsistency in the methods for melatonin assessment, difficulties in complying with the protocol of melatonin collection, and the distinctions among study designs; and (3) the erroneous premise that (lack of or excessive) melatonin could contribute or counteract (as antidepressant) depression needs to evolve to the understanding that melatonin is an integrative molecule which potentially affects mood through regulation of physiology and behavior in several levels.

Though the immediate mechanisms of action of current antidepressants are well-understood, the delay in clinical outcome conveys the gap of knowledge on the complete pathways that ultimately determine the clinical response. This delayed response shifted theories toward the neuroplastic hypothesis, whereby reduced levels of neurotrophic factors result in atrophic morphological changes, especially at the synaptic level (5). Animal studies that examine the neurobiological changes induced by experimental models that lead to depressive-like behaviors highlighted the relevance of the neuroplasticity phenomena in depression (70). The activation of cellular signaling pathways by antidepressant strategies would eventually elevate the levels of growth factors (e.g., BDNF, VEGF, VGF), promote proliferation on hippocampal progenitor cells, and ultimately restore monoaminergic synapses (70–72).

Melatonin exerts neuroprotective actions by counteracting the NMDA-mediated excitotoxic effects of glutamate, including the impaired BDNF signaling and cell death (73). Exogenous melatonin can differentially modulate glutamate release in CNS structures in rodents (74), suggesting it might correct the imbalance in the glutamatergic system in patients with mood disorders. This body of evidence suggests that melatonin may increase neuroplasticity in the CNS acting on the glutamatergic system.

### Can Urinary 6-Sulfatoxymelatonin (aMT6S) Be a Treatment Biomarker?

Biomarkers are easily accessible molecules that discriminate the presence or absence of a disease or predict treatment response. They are based on genomic, proteomic, functional, and structural features that characterize the disorders. “Diagnostic biomarkers” identifiable at any stage of the illness are often measured before treatment. The term “treatment biomarkers” refers either to baseline values that predict how effective treatments may be and guide therapeutic choice, or short-term variation between baseline and an early phase in the course of therapy (75).

A promising group of “treatment biomarkers” for SNRI/SSRI search for consistent changes in monoamines neuronal reuptake inhibition associated with subsequent mood changes (usually after 4–6 weeks). To measure the blockage of neuronal monoamine transporters directly, it would be necessary to assess changes in an immediate molecular product on the postsynaptic level that directly results from the neurotransmitter release (see section What Is the Pathway of Melatonin Production?). We proposed the assessment of 6-sulfatoxymelatonin (aMT6s, Figure 2) to detect whether the blockage of noradrenaline neuronal reuptake on the first day of medication took place. This can be done by using the relationship between the morning aMT6s content 1 day before and 1 day after the installment of the therapy. This procedure is not expected to evaluate the implication of melatonin or the pineal gland in the mechanism of action of the antidepressants, but rather to produce a proxy of the immediate events that follow the consequences of antidepressants at synaptic level. The same protocol could theoretically be used for both SNRIs and SSRIs, as the pinealocyte function depends on the uptake of serotonin, which is mediated by the transporter found in neuronal membranes.

Because the difference in aMT6s urine concentration in the light and dark phases reflects pineal activity, it has been suggested that an increase in the nighttime production of aMT6s after the first dose of antidepressant medication could work as a fingerprint of the integrity of this system; thus, it could predict a later improvement in depressive symptoms (76). A first study to test that hypothesis evaluated the association of a “aMT6-SNRI biomarker” and the improvement of depressive symptoms in MDD patients taking placebo (*n* = 12) or antidepressants (*n* = 22; fluoxetine, duloxetine or *Hypericum perforatum*) for 8 weeks. Both placebo and SNRIs groups showed improvement in depressive symptoms but only the group treated with the drug showed a significant increase in aMT6 urinary excretion in the first urine of the day (77). In patients taking clomipramine, the fraction of aMT6s excreted from 24:00 to 06:00 relative to the total amount excreted in 24 h was significantly higher 24 h after the first dose than at baseline (before treatment) in responders in comparison to non-responders (78). The same pattern was seen in 22 women taking nortriptyline (79), and 20 women taking fluoxetine (80): only responders had a significant increase in aMT6s levels. Altogether these studies suggest that the “aMT6 urinary biomarker” reveals whether the blockage of neuronal uptake was effective on the first day of administration, thus representing that aMT6 reflects the integrity of the monoaminergic systems that are required for antidepressant action. The advantage of this method over those that measure changes in the content of monoamines in leukocytes, platelets, or even plasma is the direct estimation of the increase of neurotransmission efficiency that is the inductor of neuronal plasticity.

### Do Exogenous Melatonin and Melatonin Agonists Have Therapeutic and/or Prophylactic Effects on Depression?

In a series of elegant experiments, Nagy et al. (81) showed that de 24-h pattern of the dorsal raphe 5-HT reuptake in C57BL/6J mice under shortened photoperiods may be altered at the transcriptional level by specifically timed melatonin. The data suggest that daily melatonin treatment can induce and sustain receptor 5HT1A mRNA expression throughout the light phase. In the same line, Otsuka et al. (82), using the same mice, showed that daily melatonin treatments 2 h before the end of the light phase can restore the amplitude of the daily rhythm of 5-HT contents in the amygdala. In models with higher face and construct validities, such as the chronic unpredictable mild stress (CUMS), melatonin at high concentrations (10 mg/kg) showed antidepressant-like effects, preventing the CUMS-induced decrease in norepinephrine content and the expression of tyrosine hydroxylase, dopamine-b-hydroxylase and norepinephrine transporter in the adrenal medulla (83), as well as depressive-like behaviors, such as impaired sucrose intake, physical coat deterioration, and decreased grooming (84, 85).

Overall, there is substantial heterogeneity in the body of clinical evidence regarding the prophylactic or therapeutic use of exogenous melatonin or melatonin agonists in depression, complicating a conclusive assessment. Different methodological approaches and small convenience samples precludes robust comparisons among studies, as there is no consensus on dosing and timing. Moreover, it is known that the secretion of pineal melatonin shows a wide interindividual difference as well as significant dependence on the exposure to dark each night (86); thus, therapeutic doses or an optimal serum levels are yet to be determined.

A recent systematic review (87) included eight randomized double-blind controlled trials using exogenous melatonin as an augmentation strategy in major depression, bipolar disorder or seasonal affective disorder in comparison with placebo. The dosage of exogenous melatonin ranged from 0.125 to 10 mg. Among the three studies that evaluated melatonin in the context of depression, one showed that melatonin improved subjective sleep quality but not depressive symptoms [(88), using 5–10 mg slow release melatonin], the second showed no effect [(89), using 6 mg] and the third compared the use of 3 mg slow-release melatonin plus 15 mg buspirone vs. 15 mg buspirone and placebo, showing a significant antidepressant effect of the melatonin combination (90). Of the four studies using melatonin for SAD, one (91) showed significant antidepressant effect (using 0.125 mg twice daily), while the other three did not show significant effects (92–94). Another systematic review (95) pooled results of clinical trials testing the prophylactic or therapeutic effect of melatonin for depression in adults, including comorbid conditions. Among the three studies that tested prophylactic melatonin, one study with older adults with sleep complaints showed lower depressive scores after supplementation with 5 mg melatonin at bedtime. Two other studies were in individuals with irritable bowel syndrome and found no antidepressant effect of 3 mg melatonin. Of the studies testing melatonin as a treatment for depression, one showed a decrease in depressive scores in individuals with Delayed Sleep Phase Syndrome treated with melatonin 5 mg between 19:00 and 21:00; the other six found no significant antidepressant effects. Adverse effects reported in these studies include mild sleepiness, headache, poor sleep, vivid dreams, daytime sleepiness, and fuzzy feeling.

Following the initial evidence on the potential antidepressant effect of melatonin, efforts have been directed toward the development of antidepressants with melatonin agonist activity. Among them, agomelatine (S20098, N-[2-(7-methoxynaphth-1-yl)ethyl]acetamide) first reported in 1992, is by far the best studied. This synthetic molecule has high affinity for MT1 and MT2 receptors, but also present moderate affinity for the serotonin receptor *5HT2*C. There is few clinical evidence of the effect of agomelatine in reducing overall depressive symptoms: results show agomelatine's higher response and lower remission rate compared to placebo (96, 97) and its similar efficacy compared to other antidepressants (i.e., paroxetine, fluoxetine, sertraline, escitalopram, and venlafaxine) (97, 98). However, most studies did not show superior or additional effects. Agomelatine seems to be better tolerated than other antidepressant agents, as no significant adverse events have been reported; furthermore, it did not show significant discontinuation symptoms. Some studies suggest agomelatine offers benefits for initial insomnia, improvement of sleep quality and efficiency, reduced daytime sleepiness, but no changes in sleep architecture of depressed patients (97). In one study (99), agomelatine was used in difficult-to-treat and refractory patients showing a significant improvement after 12 weeks. Of note, samples are overall composed of mild to moderate cases of depression. In addition, most studies compared agomelatine with low doses of other antidepressants. Finally, the evidence on melatonin or agomelatine efficacy in preventing or treating SAD is highly controversial (100), with no solid support for its prescription.

The knowledge of clinical use of melatonin is growing, with evidence supporting the prescription of exogenous melatonin for a few clinical situations. In the case of adult chronic insomnia, a few studies show modest benefits for sleep onset, sleep latency and total sleep time, although clinical significance is still questionable (101, 102). For this recommendation, typical doses are in the range of 1–5 mg. Melatonin is also currently recommended as an adjuvant treatment of insomnia in children and adolescents, particularly in those with comorbid ADHD or autism (103). In this case, the prescribed dose should be initially of 0.2–0.5 mg, 3–4 h prior to bedtime, with progressive increase up to 3 mg for children or 5 mg for adolescents (104).

Circadian Rhythm Sleep-Wake Disorders are a set of clinical conditions to which melatonin are classically indicated. For sleep disturbances in shift workers, the readjustment to nighttime sleep following a night work shift or the promotion of desired daytime sleep can be reached with doses of no more than 1–3 mg about 30 min prior to the desired sleep onset (105, 106). In the case of jet lag syndrome of eastward trips, exogenous melatonin can promote the adjustment of sleep phase when taken at the desired destination bedtime (107, 108). For individuals with Delayed Sleep-Wake Phase Disorder, 0.5 up to 5 mg melatonin scheduled at ~1.5–2 h prior to habitual bedtime significantly advanced sleep onset (109–111). Finally, 0.5 mg of melatonin either 1 h prior to a preferred bedtime or at a fixed time can hasten synchronization of individuals with 24-h Sleep-Wake Rhythm Disorder (107).

In conclusion, the evidence supporting the clinical use of melatonin should be analyzed very carefully. A potential therapeutic effect of melatonin for mood disorders can only be expected if compatible with the physiological regulation of melatonin detailed in this review. Pineal melatonin is a neuro-endocrine message of darkness that is physiologically secreted in humans after a few minutes in the dark, peaking shortly after, serving as an internal temporal cue to cells, tissues, and systems. Extra-pineal production of melatonin happens in a constitutive manner, and on-demand production or suppression may also be regulated by levels of glucocorticoids, and different stages of an inflammatory response triggered by immune-competent cells. Hence, the optimization of potential therapeutic uses of melatonin in psychiatric disorders must encompass the specificities of an integrative molecule inextricably linked to entrainment, metabolism, immunity, neurotransmission, and cell homeostasis.

## Conclusion and Perspectives

Localizing phenomena, dissecting underlying mechanisms, and decomposing systems to understand their functioning have been helpful strategies in building knowledge in Biology. The downside of tackling questions in such a manner is that one might overlook how interconnected systems are, and thus lose perspective of the whole. Therein lies the challenge of decomposing complex regulatory structures like the timekeeping and melatonergic systems.

Pineal melatonin is synthesized in the absence of light (typically at night) under sympathetic control. Its production relies on the neurotransmitter norepinephrine and requires serotonin as precursor, monoamines linked to depression. The rhythmic production of pineal melatonin is a message of darkness to the body, which aids in synchronizing oscillations in physiological functions and behaviors. Nevertheless, melatonin may be implicated in the pathophysiology of depressive mood for its chronobiotic effects and the various immunological processes in which it is involved. A growing number of studies highlight the relevance of exploring the potential of melatonin extra-pineal synthesis, as well as the regulation of pineal melatonin production apart from the circadian light stimuli. Therefore, the study of melatonin in the context of depression must acknowledge its regulatory effect via chronobiotic (endocrine), paracrine and autocrine functions. It is crucial to acknowledge that detectable circulating levels of melatonin are a reflection of its amplitude and phase of secretion.

Studies of the pharmacodynamics of exogenous melatonin or melatonin agonists should always anticipate distinct clinical outputs depending on the route of administration, dosage, and timing, as these substances would potentially represent (or interfere with) different physiological actions. The pleiotropic actions through which melatonin might affect mood include the role of melatonin as an internal temporal cue, as a neurohormone in close relation with the monoaminergic system, and its indirect effects on depression via the immune and stress response systems. In this context, the resulting effects of melatonin actions on mood must be understood as a more complex and multifactorial pattern of systemic regulation, rather than an “antidepressant” effect *per se*.

## Author Contributions

AT and EE reviewed and edited the final manuscript. MH was responsible for funding acquisition. All authors participated in the conceptualization and the writing of the original draft.

## Conflict of Interest

The authors declare that the research was conducted in the absence of any commercial or financial relationships that could be construed as a potential conflict of interest.
